# Exosomes released from neural progenitor cells and induced neural progenitor cells regulate neurogenesis through *miR-21a*

**DOI:** 10.1186/s12964-019-0418-3

**Published:** 2019-08-16

**Authors:** Yizhao Ma, Chunhong Li, Yunlong Huang, Yi Wang, Xiaohuan Xia, Jialin C. Zheng

**Affiliations:** 1 Center for Translational Neurodegeneration and Regenerative Therapy, Shanghai Tenth People’s Hospital affiliated to Tongji University School of Medicine, Shanghai, 200072 China; 2 Collaborative Innovation Center for Brain Science, Tongji University, Shanghai, 200092 China; 3 Departments of Pharmacology and Experimental Neuroscience, University of Nebraska Medical Center, Omaha, NE 68198-5930 USA; 4 Department of Pathology and Microbiology, University of Nebraska Medical Center, Omaha, NE 68198-5930 USA

**Keywords:** Exosome, Neural stem/progenitor cells, Induced neural stem/progenitor cells, Differentiation, *miR-21a*, Neurogenesis

## Abstract

Neural stem/progenitor cells (NPCs) are known to have potent therapeutic effects in neurological disorders through secreting exosomes. The limited numbers of NPCs in adult brain and the decline of NPC pool in many neurological disorders restrain the further use of exosomes in treating these diseases. The direct conversion of somatic cells into induced NPCs (iNPCs) provides abundant NPC-like cells to study the therapeutic effects of NPCs-originated exosomes (EXOs). Our recent study demonstrated that iNPCs-derived exosomes (iEXOs) exhibit distinct potential in facilitating the proliferation of NPCs, compared to EXOs, indicating the importance to investigate the effects of EXOs and iEXOs on the differentiation of NPCs, which remains unknown. Here, our results suggest that EXOs, but not iEXOs, promoted neuronal differentiation and neither of them had effect on glial generation. Microarray analysis revealed different miRNA signatures in EXOs and iEXOs, in which *miR-21a* was highly enriched in EXOs. Perturbation of function assay demonstrated the key roles of *miR-21a* in the generation of neurons and mediating the neurogenic potential of exosomes. Our data suggest that EXOs and iEXOs may achieve their therapeutic effects in promoting neurogenesis through transferring key miRNAs, which sheds light on the development of highly efficient cell-free therapeutic strategies for treating neurological diseases.

**Electronic supplementary material:**

The online version of this article (10.1186/s12964-019-0418-3) contains supplementary material, which is available to authorized users.

## Background

Due to the failure of clinical trial of drugs for eliminating key risk factors (e.g. Aβ) of neurodegenerative disorders, transplantation of stem cells have been considered as a promising therapeutic strategy for treating these diseases [1, 2]. Developed since 2006, the somatic reprogramming could generate a renewable source of autologous cells, which could be immune-tolerated by the recipient and overcome the ethical/religious concerns of embryonic stem cells application [3–5]. Our previous studies improved the reprogramming strategy by direct converting somatic cells (e.g. astrocytes) into induced neural stem/progenitor cells (iNPCs), which alleviated pathological features of neurodegenerative disease mouse model without teratoma formation post transplantation [6, 7].

Although the therapeutic effects of cell transplantation are well known, how do these transplanted cells exert their effects remains controversial. Due to the low survival, differentiation and integration efficiency of exogenous cells in the brain, recent findings suggested that transplanted cells might implement their therapeutic effects through secreting exosomes [8, 9]. Exogenous cells, such as mesenchymal stem cells (MSCs), could secrete exosomes, a key component of microenvironment, to promote neural plasticity and functional recovery in various central nervous system (CNS) disease models [9–11]. For example, MSCs-derived exosomes contain high levels of *miR-133b* and miRNAs in *miR-17*~*92* cluster which repress the expression of *Pten*, *CTGF* and *RhoA*, leading to neurite remodeling and functional recovery in mouse stroke models [9, 10].

As the smallest extracellular vesicles with 30~150 nm in diameter, exosomes are released from virtually all cell types in the brain. By horizontally transferring their contents including miRNAs, exosomes could regulate neurogenesis, the central part of neuroregeneration post brain injury [12–14]. Our recent studies for the first time demonstrated that iNPCs-derived exosomes (iEXOs), but not that of primary neural stem/progenitor cells (NPCs)-derived exosomes (EXOs) could promote the proliferation of NPCs in vitro, implying that iNPCs have the potential to manipulate the stem cell niche post transplantation [12]. However, the roles of reprogrammed cell-derived exosomes in neurogenesis and the underlying mechanisms remain unknown. Here, we have addressed the effects of EXOs and iEXOs on neurogenesis in vitro by co-culturing NPCs with these exosomes in defined conditions. Our results suggested that EXOs significantly promote neuronal differentiation, compared with iEXOs. Microarray analysis demonstrated distinct expression profiles of miRNAs between EXOs and iEXOs, in which *miR-21a* was highly enriched in EXOs but not iEXOs. We further identified *miR-21a* as a novel regulator of neurogliogenic commitment, which could mediate the neurogenic potential of exosomes. These results suggest potent effects of exosomes on endogenous NPCs, which shed light on the development of novel cell-free therapeutic strategies for neurological disorders.

## Methods

### Mouse NPCs isolation and enrichment

Mouse cortical NPCs were isolated from mouse fetal brain tissue as previously described [15]. Briefly, cortical tissues were isolated from embryonic day 13.5 (E13.5) mice and triturated physically 15–20 times. Dissociated tissues were filtered through 40 μm filter and single cells were cultured in substrate-free tissue culture flasks for the formation of neurospheres in NPC proliferation medium, containing NeuroCult® NSC Basal Medium (Stem Cell Technologies), NeuroCult® NSC Proliferation Supplements (Stem Cell Technologies), 20 ng/mL FGF2 (BioWalkersville), 20 ng/mL EGF (BioWalkersville) and 2 μg/mL heparin (Sigma), N2 supplement, 2 mM L-glutamine, 100 U/ml penicillin & streptomycin. Primary neurospheres were collected, centrifuged at low speed to remove flowing cells in the supernatant, dissociated into single cells with Accutase (Sigma) for 5 min, and re-plated for a second round of neurosphere formation. Enriched NPCs were harvested after three rounds of neurosphere formation.

### Differentiation of NPCs

The differentiation of NPCs and iNPCs was as previously described [7]. Briefly, 5 × 10^4^ NPCs were planted on Poly-L-Ornithine/laminin-coated coverslips in 24-well plate with DMEM/F12 supplemented with 1 × N2, 1 × B27, 1.0 mM Glutamax, 0.11 mM β-mercaptoethanol, 1.0 mM dibutyrylcAMP (Sigma), 0.2 mM ascorbic acid (Sigma), 10 ng/mL brain-derived neurotrophic factor (BDNF) (Peprotech), and 10 ng/mL glial cell line-derived neurotrophic factor (GDNF) (Peprotech) for 1–2 weeks. The medium was changed every 3 days.

### Collection of exosomes

Exosomes were isolated from the serum-free culture of NPCs as previously described [12]. Briefly, 6 × 10^6^ NPCs were plated on poly-L-Ornithine/laminin-coated 10 cm dish and cultured in NPC proliferation medium for 12 h. The supernatants were first centrifuged at 300 g for 10 min to remove flowing cells, at 3000 g for 20 min to remove cellular debris, and then at 10000 g for 30 min to remove intracellular organelles. Exosomes were collected by ultracentrifugation at 100000 g for 2 h. All centrifugation steps were carried out at 4 °C.

### miRNA mimics/inhibitors and transfection

The mimics control, *miR-21a* mimics, inhibitor control, and anti-*miR-21a* inhibitor were purchased from GenePharma (GenePharma Co., Ltd., Shanghai). Transfection of miRNA mimics/inhibitors was performed using the Lipofectamine 2000 reagent (Invitrogen) according to the manufacturer’s instruction.

### Transmission electron microscopy (TEM)

Purified exosomes were negatively stained and then spread on the copper grids. The droplets of exosomes were removed with filter paper and air-dried at room temperature. Images were obtained using transmission electron microscopy (JEM-1230, JEOL Ltd.).

### Western blot

Western blot was carried out for exosomes and cells lysates as previously described [12]. Briefly, exosomes were lysed in RIPA lysis and extraction buffer (Thermo Scientific). Protein concentration was determined using the BCA (bicinchoninic acid) Protein Assay Kit (Pierce). Blots were incubated with primary antibodies for Flotillin-1 (1:1000; BD biosciences), Flotillin-2 (1:5000; BD biosciences) and TSG101 (1:1000; Abcam) overnight at 4 °C. Corresponding HRP-conjugated anti-rabbit or anti-mouse (1:10,000, Pierce) secondary antibodies were incubated for 1 h at room temperature (RT). Bands were visualized with an ECL kit (Pierce). The density of the immunoblots was determined by image lab software and analyzed using Image J program.

### Immunocytochemistry

The cultured cells were planted on coverslips and fixed in 4% formaldehyde for 20 min at RT and then washed with PBS for three times. The fixed cells were permeabilized with 0.2% Triton X-100 in PBS for 10 min, then blocked with 2% BSA in PBS for 1 h at RT. Subsequently, they were incubated overnight at 4 °C with primary antibodies including rabbit anti-MAP 2 (1:1000; Millipore), mouse anti-βIII-Tubulin (Tuj1) (1:500; sigma) and chick anti-GFAP (1:500; Millipore). Coverslips were washed and incubated for 1 h at RT with secondary antibodies including anti-rabbit IgG (coupled with Alexa Fluor 568, Life Technologies), anti-rabbit IgG (coupled with Alexa Fluor 488, Life Technologies) and anti-chicken IgG (coupled with Alexa Fluor 488, Life Technologies), anti-mouse IgG (coupled with Alexa Fluor 488, Life Technologies). Nuclear DNA was stained with DAPI. Then the coverslips were mounted on glass slides with mounting buffer (Sigma-Aldrich). Immunostaining was examined by a Zeiss 710 confocal laser scanning microscope.

### Quantitative polymerase chain reaction

The mRNA and miRNA were isolated from cell and tissue samples using miRCURY RNA isolation kit (Exiqon, Woburn, MA). cDNA was synthesized using miScript II RT kit (Qiagen, Valencia, CA). Transcripts were amplified using Transcripts were amplified using gene-specific primer (Additional file 1: Table S1) and SYBR green PCR kit (Qiagen, Valencia, CA) with the ABI7500 (Applied Biosystems, Waltham, MA). All qPCR results measured each sample in triplicate and no-template blanks were used for negative controls. Amplification curves and gene expressions were normalized to the house-keeping gene *Gapdh* (for mRNA) and *U6* snRNA (for miRNA).

### Gene ontology analysis

Mouse *miR-21a* predicted target genes for gene ontology (GO) analyses were extracted from Targetscan.org (http://www.targetscan.org/vert_72/). DAVID bioinformatics platform (david.ncifcrf.gov/home.jsp) and Panther Classification System (http://www.geneontology.org/) were used for GO analyses. *Mus musculus* genome data was used as annotation background. Biological_Process was selected as Functional_Database for gene function classification. Minimum and maximum numbers of genes in the category were set at 2 and 1000, respectively. Benjamini & Hochberg multiple test adjustment was used to adjust *P*-value of analysis: *P*-value < 0.05 was considered a significant enriched pathway.

### Statistical analyses

All results are the means of at least three independent experiments ± SD. Data from two groups were evaluated statistically by two-tailed, paired or unpaired student *t* test. Significance was considered when *P*-value < 0.05.

## Results

### EXOs and iEXOs display differential effects on neuronal differentiation

To test the influence of EXOs and iEXOs on the differentiation of NPCs, we first collected EXOs and iEXOs using ultracentrifugation-based approach. Exosomes were visualized under transmission electron microscopy (TEM), which displayed vesicle-like structures with sizes between 30 to 150 nm (Additional file 1: Figure S1A). The size of exosomes was evaluated using Nanoparticle tracking (NTA) analysis (Additional file 1: Figure S1B). The diameter of exosomes was among the typical size arrange of exosomes, which is consistent with TEM results. Exosomes were further characterized by Western Blot for exosomes specific markers, Flotillin-1, Flotillin-2 and HSP70, confirming the purification of EXOs and iEXOs (Additional file 1: Figure S1C).

**Fig. 1:**
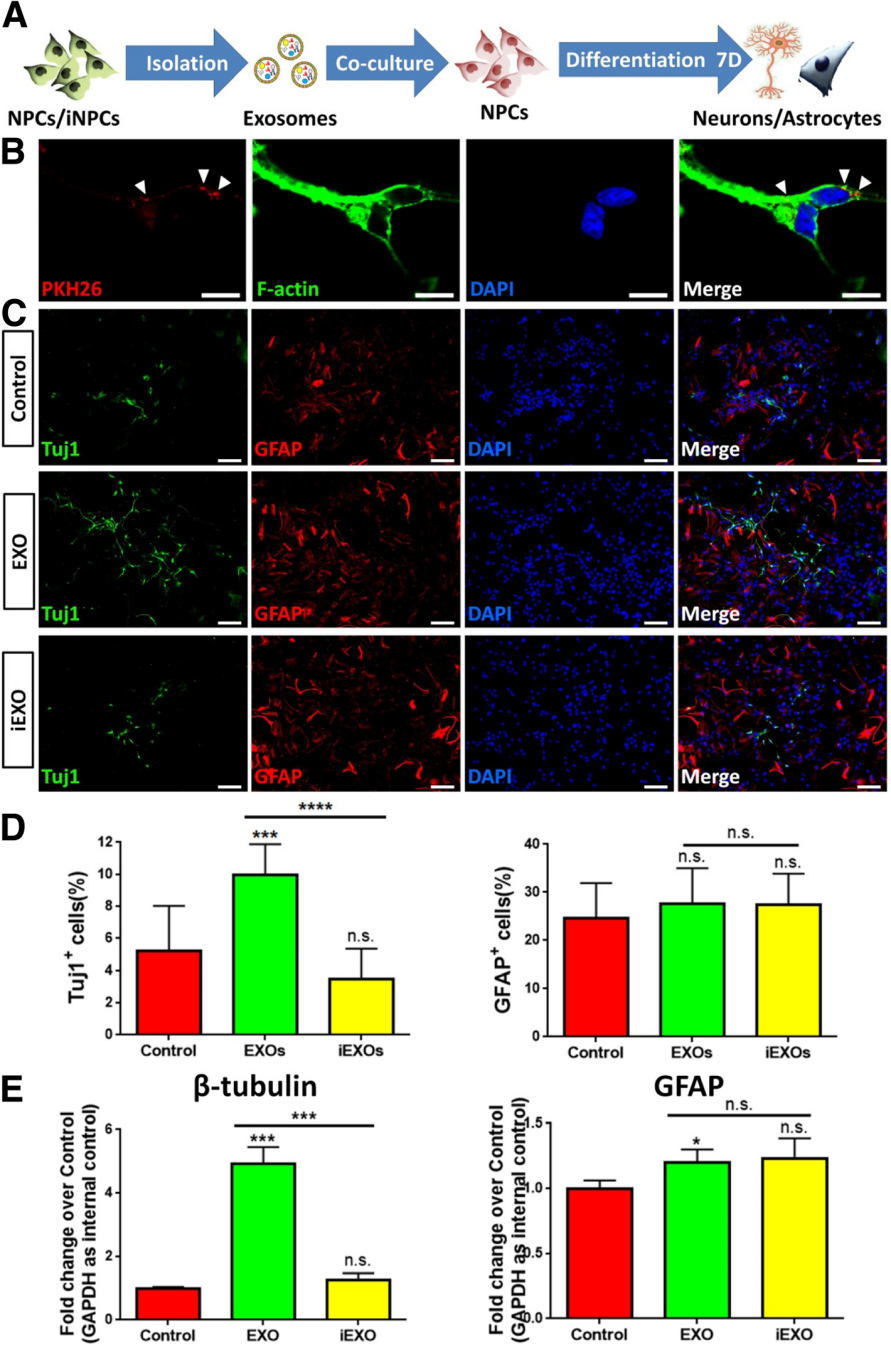
EXOs promote neuronal differentiation of NPCs. (**a**) A schematic representation of the experimental approach. (**b**) The uptake of PKH26-labeled exosomes by NPCs was determined by immunocytochemical analysis. (**c**) NPCs were co-cultured with exosomes for 7 DIV in differentiation conditions. Representative images of Tuj1 (green), GFAP (red) and DAPI (blue) staining were shown. (**d**) Quantification of Tuj1^+^ and GFAP^+^ cells (as a percentage of total cells) in the culture. (**e**) The transcript expression of *β-tubulin* and *GFAP* post exosome treatment was determined by qPCR analysis. Data were represented as mean ± SD from three independent experiments. *, *** and **** denote *p* < 0.05, *p* < 0.001 and *p* < 0.0001, respectively. n.s. denotes no significance. Scale bar 20 μm (**b**) and 100 μm (**c**)

Next, we co-cultured NPCs with either 15 μg/ml EXOs or 15 μg/ml iEXOs in differentiation conditions for 7 days in vitro (DIV) (Fig. 1a). PBS was used as control. The uptake of exosomes by NPCs was validated by co-culturing PKH26-labeled exosomes with NPCs (Fig. 1b). The immunofluorescence analysis suggested that the treatment of EXOs, but not that of iEXOs, significantly increased the proportion of Tuj1^+^ neuronal cells, compared to controls (Fig. 1c, d). In contrast, the proportions of GFAP^⁠+^ glial cells had no significant difference in both EXOs and iEXOs treated groups versus controls. The increase in the levels of transcripts corresponding to neurons (*β-tubulin*) in EXOs treated group corroborated the results, ascertained by examining the cell-type specific protein markers (Fig. 1e). To confirm our observations, we extended the culture to 14 DIV, where similar results were obtained (Additional file 1: Figure S2).

Since cells also secret soluble factors for intercellular communication, we compared the effects of EXOs with conditioned medium (CM) and exosome free CM (supernatant post-ultracentrifugation, SN) on NPCs’ differentiation (Additional file 1: Figure S3). Both immunocytochemical (Additional file 1: Figure S3A, B) and qPCR (Additional file 1: Figure S3C) analyses demonstrated that EXOs and CM had similar positive effects on neuronal differentiation, while SN had no effects, suggesting that exosomes but not soluble factors may play key roles in regulating neurogenic microenvironment. Together, our results suggested that EXOs, but not iEXOs promote neuronal differentiation, and both EXOs and iEXOs have no effect on glial differentiation.

### EXOs and iEXOs exhibit distinct miRNA profiles

**Fig. 2:**
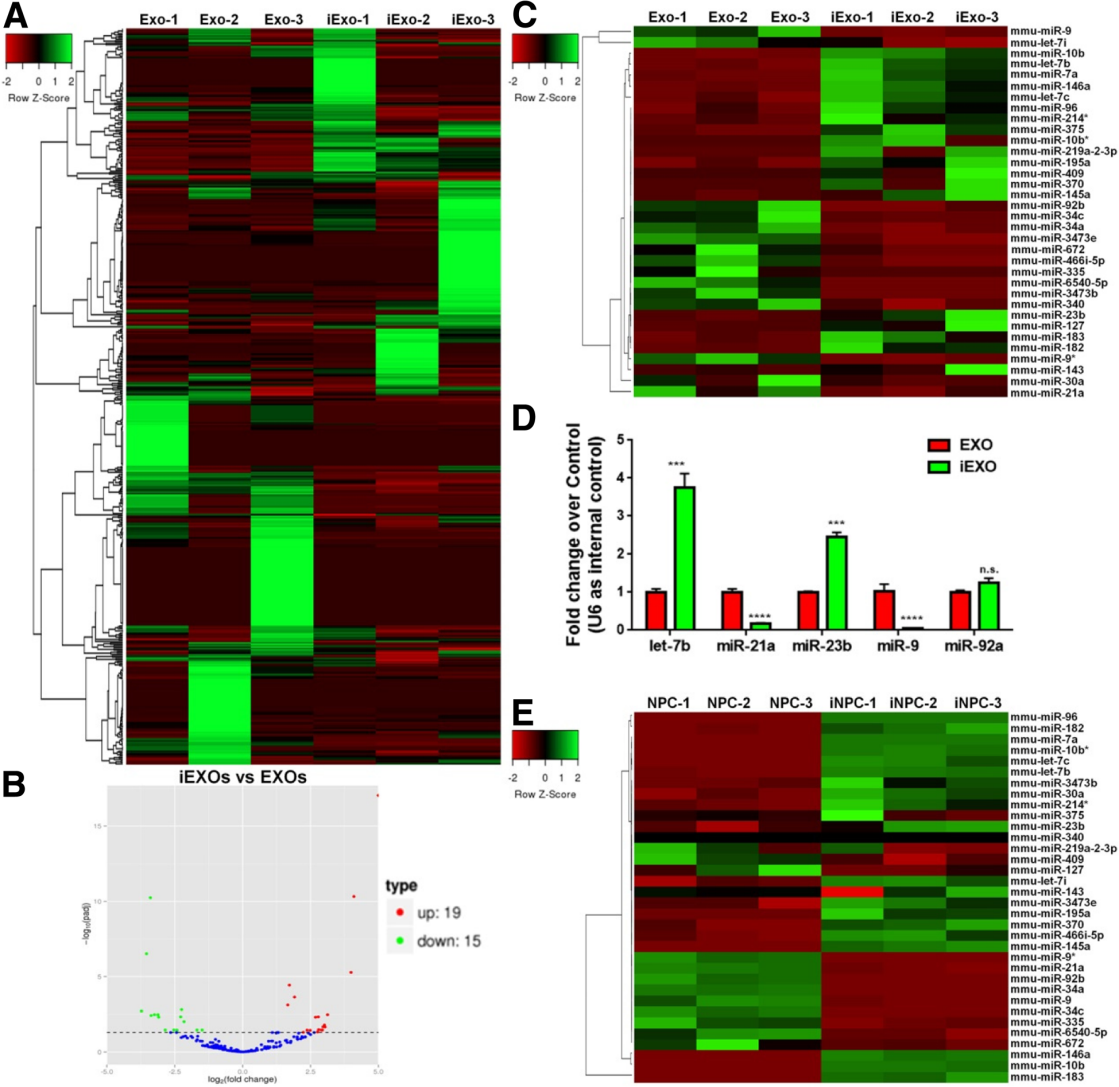
EXOs and iEXOs exhibit distinct miRNA profiles. (**a**) The miRNAs profiles of iEXOs and EXOs are represented in the heat map and hierarchical clustering-based dendrograms. (**b**) The volcano plot shows the relation between the logarithm of the *p*-values and the log fold change. (**c**) The differentially expressed miRNAs between iEXOs and EXOs are represented in the heat map and hierarchical clustering-based dendrograms. (**d**) The microarray data were validated by examining the expression patterns of randomly selected miRNAs using qPCR. (**e**) The intracellular expression levels of differentially expressed miRNAs in iNPCs and NPCs were determined by qPCR analysis and represented in the heat map. Data were represented as mean ± SD from three independent experiments. *** and **** denote *p* < 0.001 and *p* < 0.0001, respectively. n.s. denotes no significance

To understand the mechanisms underlying the differential effects of EXOs and iEXOs on neuronal differentiation, we determined their global miRNA expression profile using miRNA microarray (Fig. 2a). Of the 565 known mouse miRNAs investigated in our study, 506 were expressed in either EXOs or iEXOs. We found that the expression levels of 34 miRNAs were significantly different (fold change > 2, padj > 0.05) between EXOs and iEXOs (Fig. 2b, c). Among them, 19 miRNAs and 15 miRNAs were significantly up and down regulated, respectively, in iEXOs, compared to EXOs. A subset of miRNAs were randomly selected for qPCR analysis to corroborate miRNA microarray results (Fig. 2d). We next determined the expression levels of these 34 miRNAs in iNPCs and NPCs (Fig. 2e). 15 of 19 up-regulated miRNAs and 10 of 15 down-regulated miRNAs showed similar trends that we observed between iEXOs and EXOs, suggesting the miRNA signatures of exosomes largely inherit that of the donor cells.

### *miR-21a* regulates the cell fate commitment of NPCs

**Fig. 3:**
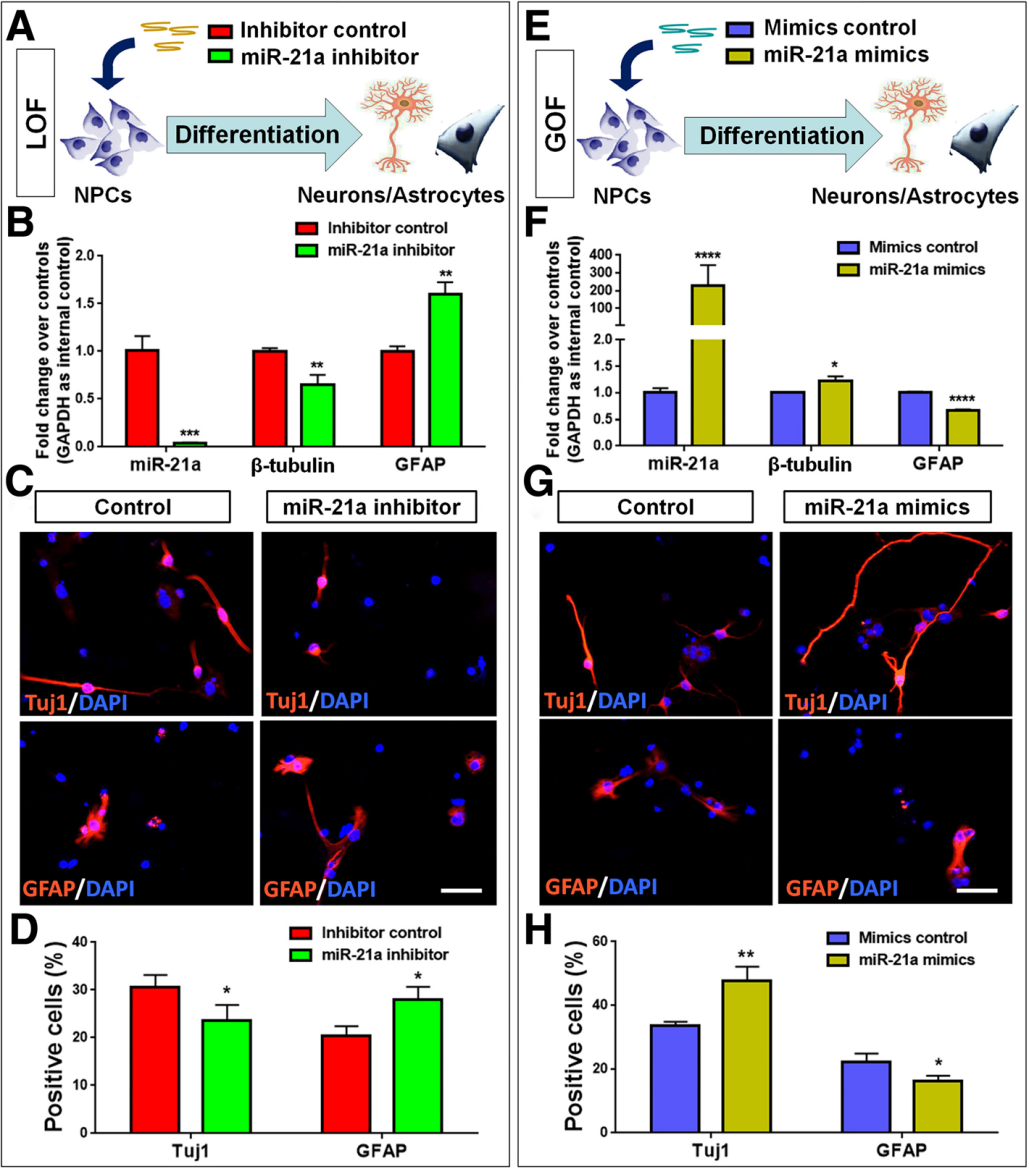
*miR-21a* regulates the neurogliogenic decision of NPCs. (**a, e**) A schematic representation of the LOF (**a**) and GOF (**e**) approaches for *miR-21a*. (**b, f**) The expression levels of *miR-21a* and transcripts corresponding to *β-tubulin* and *GFAP* were determined by qPCR. (**c, g**) NPCs were transfected with either inhibitor control/*miR-21a* inhibitors (**c**) or mimics control/*miR-21a* mimics (**g**) with exosomes for 7 DIV in differentiation conditions. Representative images of Tuj1 (red), GFAP (red) and DAPI (blue) staining were shown. (**d, h**) Quantification of Tuj1^+^ and GFAP^+^ cells (as a percentage of total cells) in the culture. Data were represented as mean ± SD from three independent experiments. *, **, *** and **** denote p < 0.05, *p* < 0.01, p < 0.001 and p < 0.0001 in comparison to control, respectively. Scale bar 100 μm (**c, g**)

Interestingly, our screening approach unveiled that *miR-21a* levels were significantly lower in iEXOs and iNPCs, compared to EXOs and NPCs, respectively. Previous studies have reported that *miR-21* is involved in the modulation of various cellular processes, especially in stem/progenitor cells [16–19]. Our qPCR results suggested that miR-21a levels increase with time during brain development (Additional file 1: Fig. S4A) and NPCs’ differentiation (Additional file 1: Figure S4B), implying its role in regulating neurogenesis. In order to determine whether *miR-21a* mediates the differential effects of iEXOs and EXOs on neuronal differentiation, we first examined the roles of *miR-21a* in NPCs' differentiation by loss-of-function (LOF) and gain-of-function (GOF) approaches using *miR-21a* specific inhibitor and mimics, respectively. NPCs were firstly transfected with either *miR-21a* inhibitor (=LOF group) or inhibitor control and cultured in differentiation conditions for 7 DIV (Fig. 3a). The efficiency of transfection is validated by qPCR, where significant down-regulation of *miR-21a* expression levels were observed in the *miR-21a* LOF group, compared to controls (Fig. 3b). qPCR analysis also revealed a significant decrease and increase in the expression levels of *β-tubulin* and *GFAP*, respectively, in the *miR-21a* LOF group versus controls (Fig. 3b). qPCR results were corroborated by immunofluorescence analysis. The proportion of Tuj1^+^ cells reduced significantly while that of GFAP^+^ cells significantly increased when *miR-21a* expression was inhibited (Fig. 3c, d). Next, NPCs were transfected with either *miR-21a* mimics (=GOF group) or mimics control and cultured in differentiation conditions for 7 DIV (Fig. 3e). In contrast to the LOF approach, the ectopic expression of *miR-21a* significantly promoted *β-tubulin* expression and repressed *GFAP* expression (Fig. 3f). Meanwhile, the proportion of Tuj1^+^ cells increased significantly while that of GFAP^+^ cells decreased significantly in the *miR-21a* LOF group versus controls (Fig. 3g, h). Therefore, both LOF and GOF studies demonstrated that *miR-21a* regulates the cell fate commitment of NPCs by facilitating neurogenesis and inhibiting gliogenesis.

### *miR-21a* mediates the differential effects of EXOs and iEXOs on neuronal differentiation

**Fig. 4:**
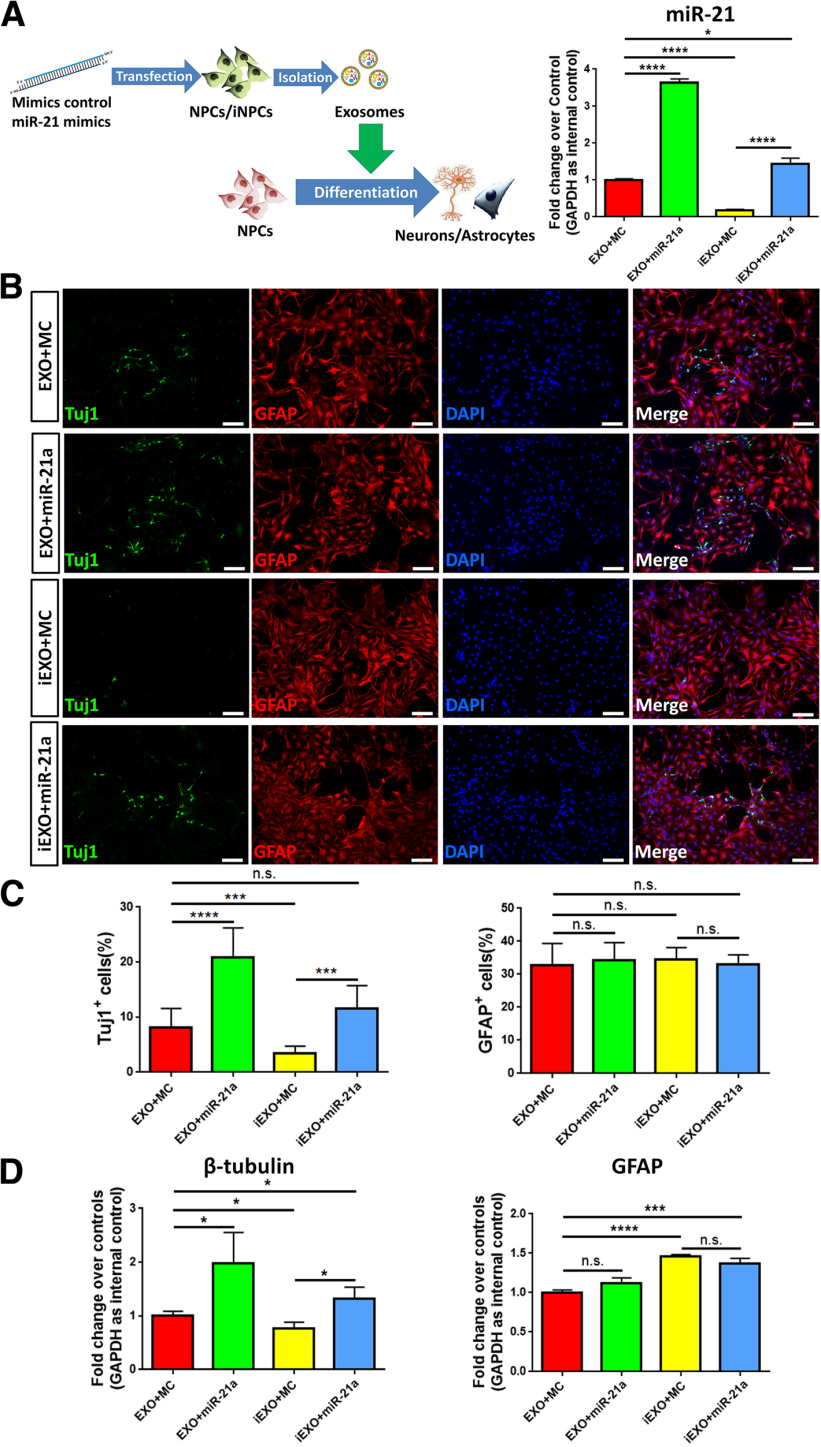
*miR-21a* mediates the effects of exosomes on neuronal differentiation. (**a**) A schematic representation of the experimental approach (left panel). The overexpression of *miR-21a* in iEXOs and EXOs was examined by qPCR analyses (right panel). (**b**) NPCs were co-cultured with control or *miR-21a* overexpressed exosomes for 7 DIV in differentiation conditions. Representative images of Tuj1 (green), GFAP (red) and DAPI (blue) staining were shown. (**c**) Quantification of Tuj1^+^ and GFAP^+^ cells (as a percentage of total cells) in the culture. (**d**) The transcript expression of *β-tubulin* and *GFAP* in differentiated NPCs after co-culturing with control or *miR-21a* overexpressed exosomes was determined by qPCR analysis. MC, mimics control. Data were represented as mean ± SD from three independent experiments. *** and **** denote *p* < 0.001 and *p* < 0.0001, respectively. n.s. denotes no significance. Scale bar 100 μm (**b**)

To determine whether the differential effects of EXOs and iEXOs on neuronal differentiation is caused by the distinct levels of *miR-21a* between these two types of exosomes, we transfected NPCs and iNPCs with either *miR-21a* mimics or mimics control using the approach described above and collected exosomes in the culture medium 48 h post transfection (Fig. 4a). qPCR analysis revealed that the expression of exosomal *miR-21a* was significantly increased when *miR-21a* was overexpressed in NPCs and iNPCs. NPCs were then co-cultured with the respective exosomes, with or without *miR-21a* overexpression, under differentiation conditions for 7 DIV. The immunofluorescence analysis demonstrated that the neuronal differentiation was significantly promoted when *miR-21a* was overexpressed in EXOs and iEXOs, while the glial differentiation remained unchanged, determined by the quantification of Tuj1^+^ and GFAP^+^ (Fig. 4b, c). The similar proportions of Tuj1^+^ cells between EXOs and iEXOs with *miR-21a* overexpression suggested that the less neurogenic potential of iEXOs could be offset by complementing the low levels of *miR-21a* in iEXOs. Our observations were confirmed by qPCR analysis that *miR-21a*-overexpressed exosomes had higher capacity to promote neuronal differentiation, ascertained by significant increases in *β-tubulin* transcript levels in *miR-21a* overexpression groups, compared with control groups (Fig. 4d). Together, our results suggested that *miR-21a* could mediate the differential effects of EXOs and iEXOs on neuronal differentiation.

## Discussion

Recent studies demonstrated that exosomes secreted from NPCs can achieve similar therapeutic effect as NPCs transplantation in treating CNS disorders, such as stroke [20, 21]. Compared to NPCs transplantation, the administration of exosomes avoids the risk of teratoma formation, autoimmune response, and religious/ethical concerns. Besides, exosomes have been considered as an excellent carrier for drug delivery for the treatment of neurodegenerative diseases due to its unique physical/chemical characteristics [22–24]. Thus, exosome-based cell-free therapeutic strategy has received growing interest within the scientific community. However, the effects of reprogrammed NPCs-derived exosome on endogenous neurogenesis remain largely unknown. Our recent findings suggest that iEXOs could promote the proliferation of NPCs by activating MEK-ERK signaling pathway, the first time demonstrating that iNPCs could regulate NPCs through secreting exosomes [12]. This work is the follow-up study to examine the effects of EXOs and iEXOs on the differentiation capacity of NPCs. We observed that though both types of exosomes have no effects on glial differentiation, EXOs, but not iEXOs could promote the generation of neurons from NPCs. Recently, Takeda and Xu reported that differentiating P12 neuronal cells release exosomes which could promote neuronal differentiation of human MSCs [25]. Surprisingly, our observations revealed that even cultured in proliferation conditions, NPCs could release exosomes with neurogenic potential. One possibility is that the NPCs used in our studies were isolated from mouse cortical tissue at embryonic day 14, when robust neurogenesis takes place in vivo. Though cultured in growth factors-containing medium to promote proliferation, those NPCs retain the innate “developmental program” which preserves the neurogenic microenvironment by releasing exosomes that promote neuronal differentiation. As gliogenesis generally initiates since postnatal stage, mouse embryonic NPCs do not release exosomes with high gliogenic potential. It also explains the reason why iEXOs exhibit no potential to facilitate neurogenesis since this innate “developmental program” is missing. Since iEXOs significantly accelerate the proliferation of NPCs, the administration of iEXOs and EXOs successively may promote the expansion of endogenous NPCs pool and then facilitate neuronal differentiation to overcome the lack of neuroregenerative capacity of brain tissues in neurodegenerative disorders, which will be examined in our future work.

The difference between NPCs and iNPCs leads to distinct expression patterns of intracellular miRNAs, which influences the miRNA contents of exosomes released from these cells, such as *miR-21a*. To date, multiple mechanisms have been reported to regulate *miR-21a* expression. REST, the proneural gene transcriptional repressor, could negatively regulate *miR-21* levels in embryonic stem cells to maintain the pluripotency [19]. During CNS development, REST needs to be repressed to preserve neurogenesis and then up-regulated postnatally to initiate the generation of glial cells, especially astrocytes. We found iNPCs inherit the characteristics of their donor cells, astrocytes, with high REST expression levels (Additional file 1: Figure S5), which, could repress *miR-21* expression. It explains the less neurogenic potential of iNPCs and lower levels of *miR-21* in iEXOs, compared to NPCs and EXOs, respectively. Other transcription repressors, such as Gfi1, could also bind to *miR-21* loci to inhibit its expression, but no difference in Gfi1 expression levels was observed between iNPCs and NPCs [26] (Additional file 1: Figure S5). Besides, TGFβ and Her2 signaling pathways were reported to promote *miR-21* expression, which enhances cancer cell invasion and renal fibrosis [27, 28]. However, our data indicated TGFβ and Her2 signaling pathways were either more active or with similar activities in iNPCs versus NPCs (Additional file 1: Figure S5), excluding their involvement in *miR-21* regulation.

The distinct signatures of exosomal miRNAs could explain, partially at least, the different functions of iEXOs and EXOs in the regulation of NPCs. For example, *miR-9* and miR-9*, which levels are higher in EXO than iEXOs, is highly involved in neurogenesis [29, 30]. And *miR-96*, which is highly enriched in iEXOs, is to promote proliferation of various cell types [31, 32]. The roles of *miR-21* in neurogenesis were recently investigated using rat NPCs [16, 17]. In defined conditions, *miR-21* could promote the generation of neurons by activating Akt and Wnt signaling pathways. Our data matched with others’ observations to identify *miR-21a* as an important regulator of neuronal differentiation. More importantly, *miR-21a* is highly enriched in exosomes and mediates the cellular functions of the latter. Gene Ontology (GO) analysis determined that the predicted targets of *miR-21* are highly enriched in biological processes (BPs) like “nervous system development” and “cell differentiation” (Additional file 1: Figure S6A). Among genes in these BPs, the expression levels of *Sox2*, *Stat3*, *Cd47* and *Bcl2* were negatively correlated with that of *miR-21a* (Additional file 1: Figure S6B, C). Hence, the profound influence of *miR-21a* on neuronal differentiation is likely due to its unique position in the regulatory hierarchy, as some of the most prominent targets of *miR-21a* are transcripts corresponding to key NPCs regulators, such as *Sox2* and *Stat3* [33, 34]. Additionally, the levels of exosomal *miR-21a* may negatively influence Bcl2 expression in recipient cells, which regulates apoptosis. This premise is confirmed by co-culture studies that iEXOs exhibit higher capacity in promoting NPCs’ survival than EXOs due to the activation of Bcl2 signaling (data not shown). Thus, the horizontally transferring of *miR-21a* from parent cells to recipient cells through exosomes could lead to the decline of these genes’ expression, which is required for adversely affecting NPCs maintenance and facilitating neuronal differentiation.

## Conclusions

In summary, our study demonstrated the distinct effects of iEXOs and EXOs on neuronal differentiation, implying the importance of exosomes in the modification of microenvironment. The microarray analysis and perturbation of function assay further identified *miR-21a* as a key factor in mediating the functions of exosomes. It is the first evidence demonstrating that NPCs and iNPCs may exhibit distinct influence in modifying the microenvironment in favor to neurogenesis, through secretion of exosomes with unique miRNAs signatures. Thus, our study, combining with our previous reports, provides a possible mechanism for the therapeutic effects of NPCs/iNPCs transplantation, shedding light on the development of exosome-based therapeutic strategies to expand the endogenous NPCs population and activate neurogenesis in vivo.

## Additional file

Additional file 1: Supplement figures and materials. (DOCX 1393 kb)

## Data Availability

The datasets used and/or analyzed during the current study are available from the corresponding authors on reasonable request.

## Abbreviations

BPs: Biological processes
CNS: Central nervous system
DIV: Days in vitro
EXOs: NPCs-derived exosomes
GO: Gene ontology
GOF: Gain of function
iEXOs: iNPCs-derived exosomes
iNPCs: Induced neural stem/progenitor cells
LOF: Loss of function
MSCs: Mesenchymal stem cells
NPCs: Neural stem/progenitor cells
NTA: Nanoparticle tracking
TEM: Transmission electron microscopy
