# Supplementary material for: Exosomes released from neural progenitor cells and induced neural progenitor cells regulate neurogenesis through miR-21a
Source: Cell Commun Signal. 2019 Aug 16;17:96. doi: 10.1186/s12964-019-0418-3 (PMC6698014; doi:10.1186/s12964-019-0418-3)
Supplement: Supplementary file 1 — Supplement figures and materials. (DOCX 1393 kb) [file 12964_2019_418_MOESM1_ESM.docx]

**Supplemental Information**

**Exosomes released from neural progenitor cells and induced neural progenitor cells regulate neurogenesis through miR-21a**

**Yizhao Ma, Chunhong Li, Yunlong Huang, Xiaohuan Xia, Jialin C. Zheng**

**Supplemental Materials**

Supplementa Figure 1

Supplemental Figure 2

Supplemental Figure 3

Supplemental Figure 4

Supplemental Figure 5

Supplemental Figure 6

Supplemental Table 1

**
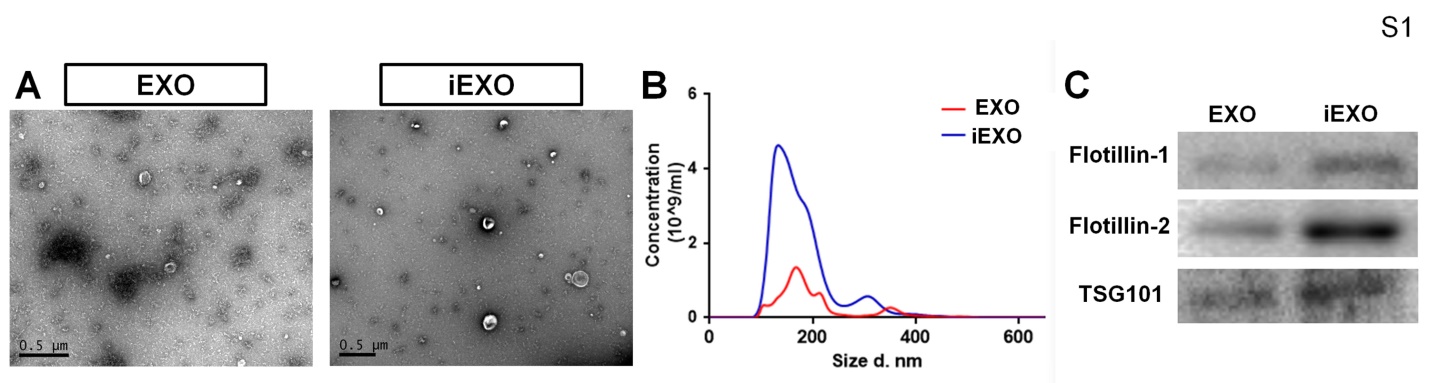
**

**Figure S1. Characterization of exsomes released from NPCs and iNPCs.**

(**A**) Exosomes were isolated from supernatants culture with NPCs and iNPCs for 12 h and observed under Transmission electron microscopy (TEM) using negative staining. (**B**) The size of exosomes derived from NPCs/iNPCs was assessed by NTA analysis. (**C**) Protein lysates were prepared from NPCs- and iNPCs-derived exomes. The levels of exosome markers Flotillin-1, Flotillin-2, and HSP70 were determined by Western blots. Scale bar, 500 nm (**A**). Experiments were carried out three times in triplicates.

**
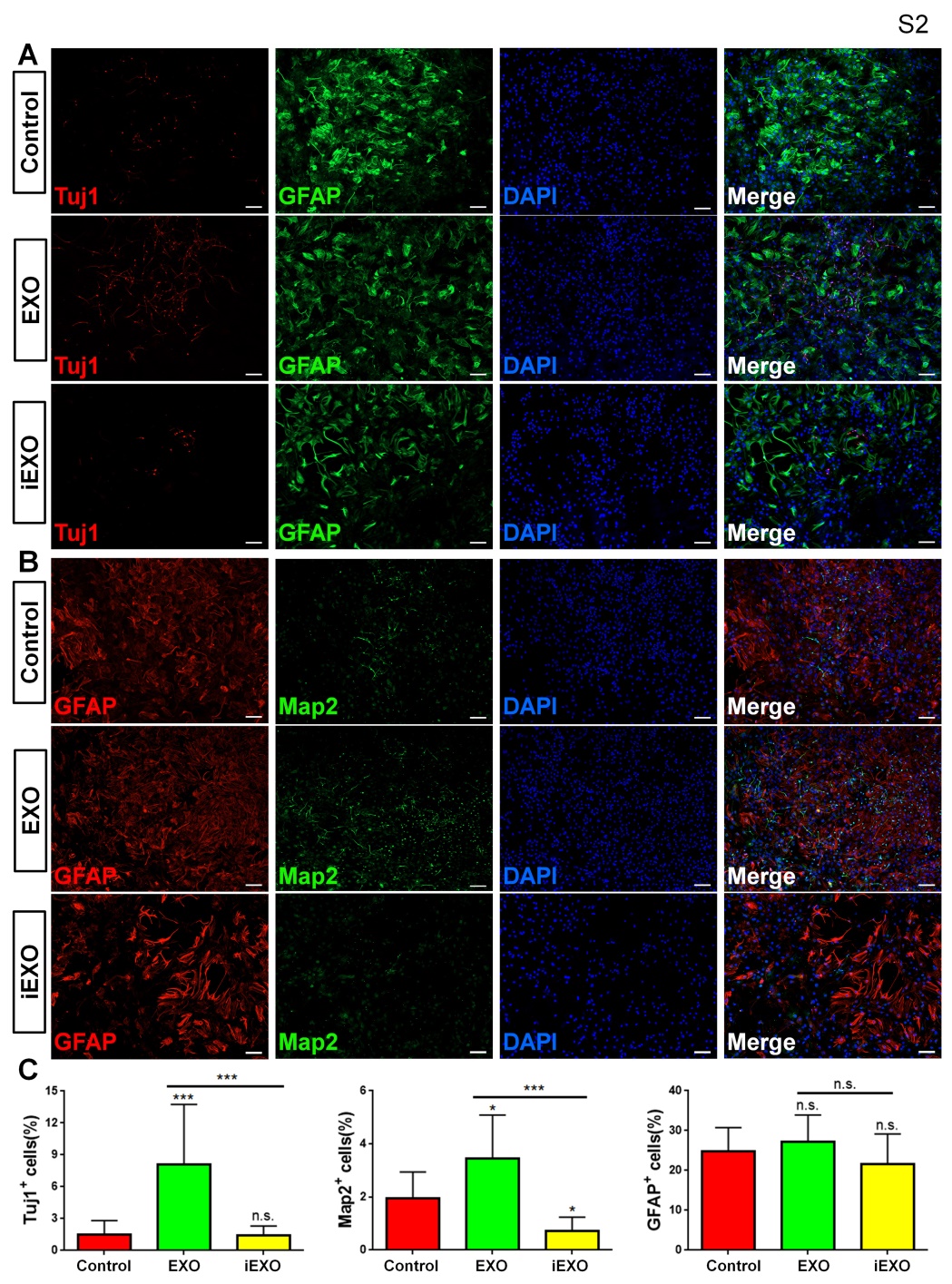
**

**Figure S2. Effects of EXOs/iEXOs on long-term differentiation of NPCs.**

(**A**) Representative immunofluorescent images showing expression of Tuj1 (in red), GFAP (in green) and DAPI (in blue) in NPCs cultured in differentiation conditions for 14 days. (**B**) Representative immunofluorescent images showing expression of GFAP (in red), Map2 (in green) and DAPI (in blue) in NPCs cultured in differentiation conditions for 14 days. (**C**) Quantification of Tuj1^+^/Map2^+^/GFAP⁠^+^ cells (as a percentage of total cells) in the culture. Scale bar, 100 μm (**A, B**). Data are mean ± SD. ∗∗∗p < 0.001 and ∗p < 0.05. n.s. denotes no significance. Experiments were carried out three times in triplicates.


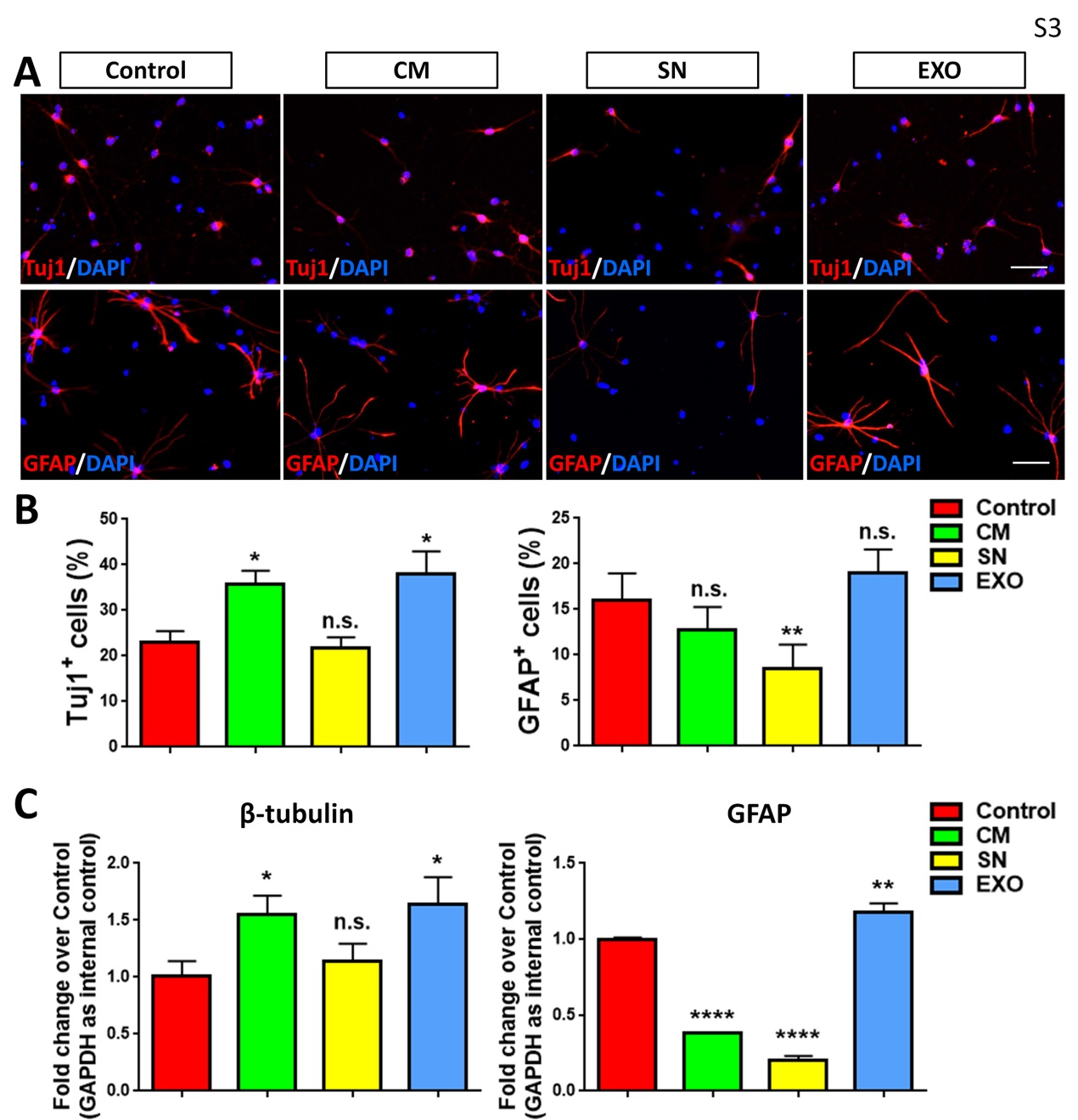


**Figure S3. Exosomes are the key components of conditioned medium on neuronal differentiation regulation.**

(**A**) Representative immunofluorescent images showing expression of Tuj1 and GFAP in NPCs cultured in defined conditions for differentiation. (**B**) Quantification of Tuj1^+^ and GFAP⁠^+^ cells (as a percentage of total cells) in the culture. (**C**) The transcript expression of *β-tubulin* and *GFAP* was determined by qPCR analysis. Scale bar, 50 μm (**A**). Data are mean ± SD. ∗∗∗∗p < 0.0001, ∗∗p < 0.01 and ∗p < 0.05. n.s. denotes no significance. Experiments were carried out three times in triplicates. CM, conditioned medium; SN, exosome-free supernatant; EXO, exosomes.


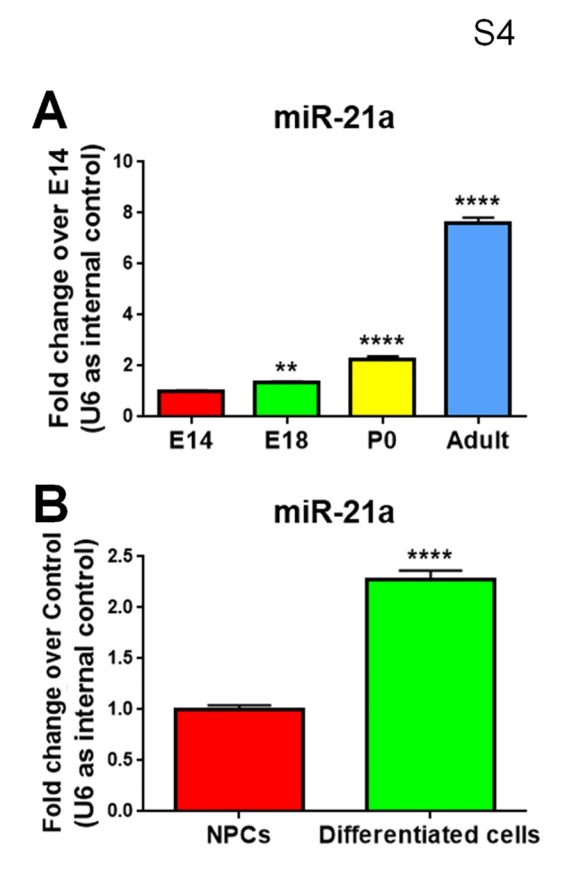


**Figure S4. Temporal expression patterns of *miR-21a* *in vivo* and *in vitro*.**

(**A**) qPCR analysis of *miR-21a* expression patterns during brain development. (**B**) qPCR analysis of *miR-21a* expression levels in NPCs and differentiated cells. Data are mean ± SD. ∗∗∗∗p < 0.0001, ∗∗p < 0.01. Experiments were carried out three times in triplicates.


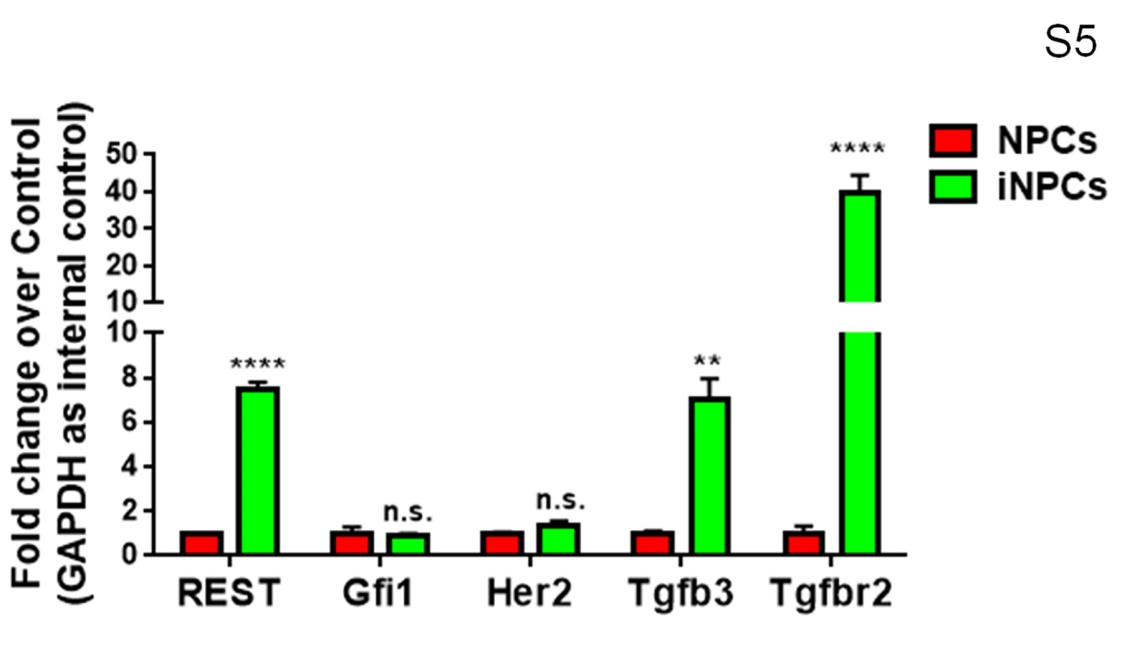


**Figure S5. Expression of *miR-21* up-stream regulators.**

qPCR analysis of candidate *miR-21a* up-stream regulators in NPCs and iNPCs. Data are mean ± SD. ∗∗∗∗p < 0.0001, ∗∗p < 0.01. n.s. denotes no significance. Experiments were carried out three times in triplicates.

**
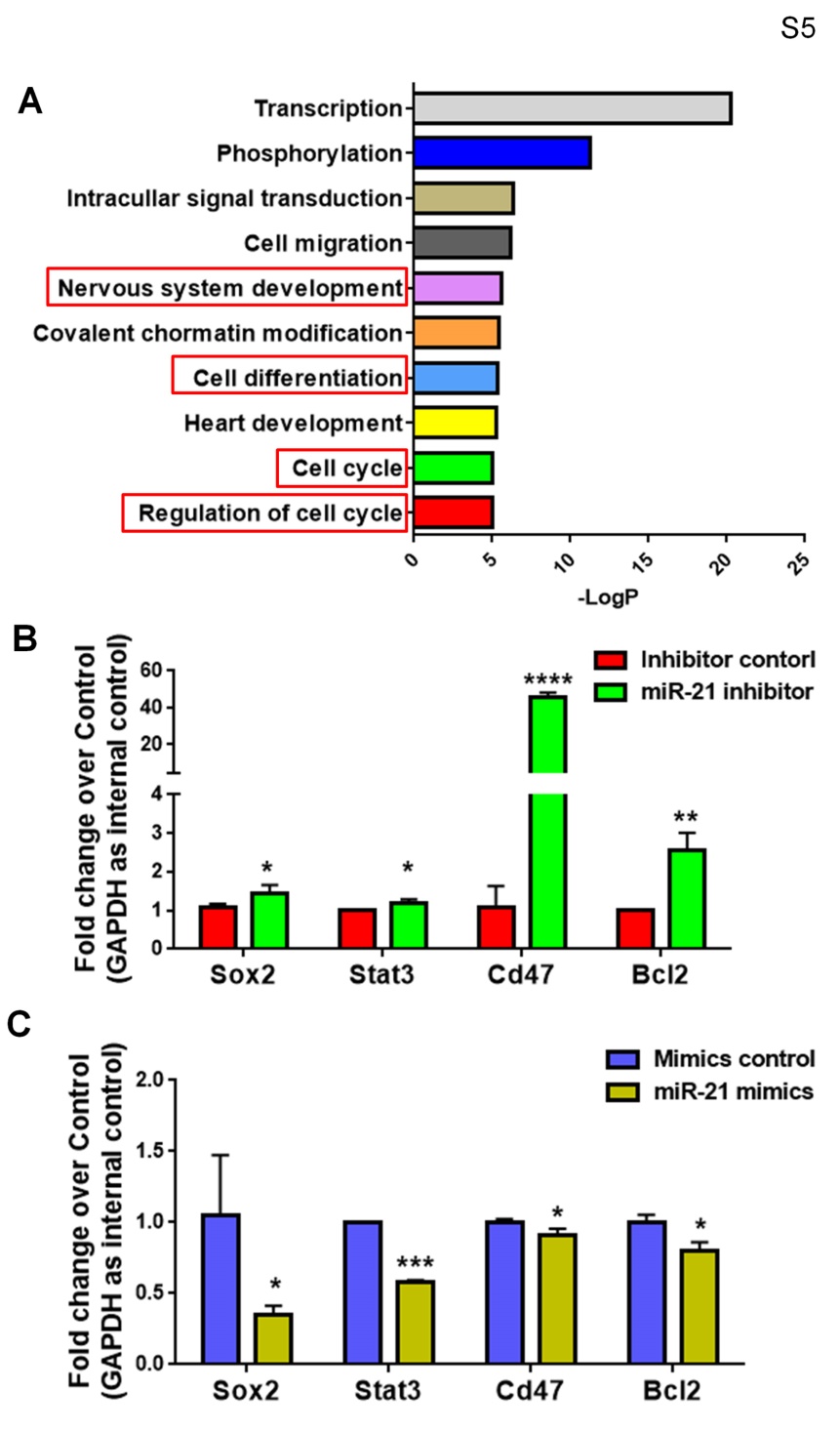
**

**Figure S6. *miR-21a* targets prediction.**

(**A**) The GO analysis of top 10 enriched biological processes in the predicted *miR-21a* target genes. (**B, C**) qPCR analysis of candidate *miR-21a* target genes in the *miR-21a* LOF (**B**) and GOF (**C**) groups, compared to their respective controls. Data are mean ± SD. ∗∗∗∗p < 0.0001, ∗∗∗p < 0.001, ∗∗p < 0.01, and ∗p < 0.05. Experiments were carried out three times in triplicates for *in vitro* perturbation.

| Gene | Sequence | Size(bp) | T^o^ | Accession N. |
| --- | --- | --- | --- | --- |
| *β-tubulin*  *(Tuj1)* | 5’-CTTTATCTTCGGTCAGAGTGGTGC-3’  5’-TTCTTTCCGCACGACATCTAGG-3’ | 103 | 57 | NM_023279.2 |
| *Bcl2* | 5’-CTGGCATCTTCTCCTTCCAGC-3’  5’-ACCTACCCAGCCTCCGTTATC-3’ | 446 | 56 | NM_177410.3 |
| *Cd47* | 5’-AGGGGCAAACGTTTCAGGGG-3’  5’-GCAAAGCTGCTGGCAACCTG-3’ | 192 | 57 | NM_010581.3 |
| *Her2* | 5’-CTTCGGGTCATTCGGGGACG-3’  5’-CCACGGGCACACAGTGAGTT-3’ | 272 | 57 | NM_001003817.1 |
| *GAPDH* | 5’-CATGTTCCAGTATGACTCCACTC-3’  5’-GGCCTCACCCCATTTGATGT-3’ | 136 | 60 | NM_001289726.1 |
| *GFAP* | 5’-TTGCTGGAGGGCGAAGAAAA-3’  5’-CATCCCGCATCTCCACAGTC-3’ | 148 | 57 | NM_010277.3 |
| *Gfi1* | 5’-CATCCACTCGGACACTCGGC-3’  5’-CCCTTGGTGCACAGCTCACA-3’ | 216 | 57 | NM_008114.3 |
| *REST* | 5’-GTGCGAACTCACACAGGAGA-3’  5’-AAGAGGTTTAGGCCCGTTGT-3’ | 201 | 52 | NM_011263.2 |
| *Sox2* | 5’-AGTCTCCAAGCGACGAAAAA-3’  5’-GCAAGAAGCCTCTCCTTGAA-3’ | 141 | 57 | NM_003106 |
| *Stat3* | 5’-CCCCGTACCTGAAGACCAAG-3’  5’-TCCTCACATGGGGGAGGTAG-3’ | 159 | 57 | NM_011486.5 |
| *Tgfb3* | 5’-AGAGGGCCCTGGACACCAAT-3’  5’-CTGCGGAGGTATGGGCAAGG-3’ | 163 | 57 | NM_009368.3 |
| *Tgfbr2* | 5’-TGCCGCTTCTCCCAAGTGTG-3’  5’-TCGGGACTGCTGGTGGTGTA-3’ | 132 | 57 | NM_009371.3 |
| miRNA |  |  |  |  |
| Universal primer | 5’-GAATCGAGCACCAGTTACGC-3’ |  |  |  |
| *U6* | 5’-TGGCCCCTGCGCAAGGATG-3’ |  | 55 |  |
| *let-7b* | 5’-TGAGGTAGTAGGTTGTGTGGTT-3’ |  | 55 | MIMAT0000522 |
| *let-7c* | 5’-TGAGGTAGTAGGTTGTATGGTT-3’ |  | 55 | MIMAT0000776 |
| *let-7i* | 5’-TGAGGTAGTAGTTTGTGCTGTT-3’ |  | 55 | MIMAT0000415 |
| *miR-10b* | 5’-TACCCTGTAGAACCGAATTTGTG-3’ |  | 55 | MIMAT0000208 |
| *miR-10b** | 5’-CAGATTCGATTCTAGGGGAATA-3’ |  | 55 | MIMAT0004538 |
| *miR-127* | 5’- TCGGATCCGTCTGAGCTTGGCT-3’ |  | 55 | MIMAT0000139 |
| *miR-143* | *5’-*TGAGATGAAGCACTGTAGCTC*-3’* |  | 55 | MIMAT0000247 |
| *miR-145a* | *5’-*GTCCAGTTTTCCCAGGAATCCCT*-3’* |  | 55 | MIMAT0000157 |
| *miR-146a* | *5’-*TGAGAACTGAATTCCATGGGTT*-3’* |  | 55 | MIMAT0000158 |
| *miR-182* | 5’-TTTGGCAATGGTAGAACTCACACCG-3’ |  | 55 | MIMAT0000211 |
| *miR-183* | 5’-TATGGCACTGGTAGAATTCACT-3’ |  | 55 | MIMAT0000212 |
| *miR-195a* | 5’-TAGCAGCACAGAAATATTGGC-3’ |  | 55 | MIMAT0000225 |
| *miR-21a* | *5’-*TAGCTTATCAGACTGATGTTGA*-3’* |  | 55 | MIMAT0000530 |
| miR-214* | 5’-TGCCTGTCTACACTTGCTGTGC-3’ |  | 55 | MIMAT0004664 |
| miR-219a-2 | 5’-AGAATTGTGGCTGGACATCTGT-3’ |  | 55 | MIMAT0022841 |
| *miR-23b* | *5’-*ATCACATTGCCAGGGATTACC*-3’* |  | 55 | MIMAT0000125 |
| miR-30a | 5’-TGTAAACATCCTCGACTGGAAG-3’ |  | 55 | MIMAT0000128 |
| miR-335 | 5’-TCAAGAGCAATAACGAAAAATGT-3’ |  | 55 | MIMAT0000766 |
| miR-34a | 5’-TGGCAGTGTCTTAGCTGGTTGT-3’ |  | 55 | MIMAT0000542 |
| miR-34c | 5’-AGGCAGTGTAGTTAGCTGATTGC-3’ |  | 55 | MIMAT0000381 |
| miR-340 | 5’-TTATAAAGCAATGAGACTGATT-3’ |  | 55 | MIMAT0004651 |
| miR-3473b | 5’-GGGCTGGAGAGATGGCTCAG-3’ |  | 55 | MIMAT0020367 |
| miR-3473e | 5’- GGGCTGGAGAGATGGCTCGTA-3’ |  | 55 | MIMAT0025587 |
| miR-370 | 5’-GCCTGCTGGGGTGGAACCTGGT-3’ |  | 55 | MIMAT0001095 |
| miR-375 | 5’-TTTGTTCGTTCGGCTCGCGTGA-3’ |  | 55 | MIMAT0000739 |
| miR-409 | 5’-GAATGTTGCTCGGTGAACCCCT-3’ |  | 55 | MIMAT0001090 |
| miR-466i-5p | 5’-TGTGTGTGTGTGTGTGTGTG-3’ |  | 55 | MIMAT0017325 |
| miR-6540-5p | 5’-CTAAGGCAGGCAGACTTCAGTG-3’ |  | 55 | MIMAT0025585 |
| miR-672 | 5’-TGAGGTTGGTGTACTGTGTGTGA-3’ |  | 55 | MIMAT0003735 |
| miR-7a | 5’-TGGAAGACTAGTGATTTTGTTGT-3’ |  | 55 | MIMAT0000677 |
| *miR-9* | *5’-*TCTTTGGTTATCTAGCTGTATGA*-3’* |  | 55 | MIMAT0000142 |
| miR-9* | 5’-ATAAAGCTAGATAACCGAAAGT-3’ |  | 55 | MIMAT0000143 |
| miR-92a | 5’-TATTGCACTTGTCCCGGCCTG-3’ |  | 55 | MIMAT0000539 |
| miR-96 | 5’-TTTGGCACTAGCACATTTTTGCT-3’ |  | 55 | MIMAT0000541 |

**Supplemental table 1. List of specific primers.**
